# Resource allocation for biomedical research: analysis of investments by major funders

**DOI:** 10.1186/s12961-020-0532-0

**Published:** 2020-02-17

**Authors:** Ambinintsoa H. Ralaidovy, Taghreed Adam, Philippe Boucher

**Affiliations:** 1 Science Division, World Health Organization, Avenue Appia 20, 1211, Geneva 27, Switzerland; 2 Data, Analytics and Delivery for Impact Division, World Health Organization, Geneva, Switzerland

**Keywords:** Funding, Investments, Health research and development, Global

## Abstract

**Background:**

Data on grants for biomedical research by 10 major funders of health research were collected from the World RePORT platform to explore what is being funded, by whom and where. This analysis is part of the World Health Organization Global Observatory on Health Research and Development’s work with the overall aim to enable evidence-informed deliberations and decisions on new investments in health research and development. The analysis expands on the interactive data visualisations of these data on the Observatory’s website and describes the methods used to enable the categorisation of grants by health categories using automated data-mining techniques.

**Methods:**

Grants data were extracted from the World RePORT platform for 2016, the most recent year with data from all funders. A data-mining algorithm was developed in Java to categorise grants by health category. The analysis explored the distribution of grants by funder, recipient country and organisation, type of grant, health category, average grant duration, and the nature of collaborations between recipients of direct grants and the institutions they collaborated with.

**Results:**

Out of a total of 69,420 grants in 2016, the United States of America’s National Institutes of Health funded the greatest number of grants (52,928; 76%) and had the longest average grant duration (6 years and 10 months). Grants for research constituted 70.4% (48,879) of all types of grants, followed by grants for training (13,008; 18.7%) and meetings (2907; 4.2%). Of grant recipients by income group, low-income countries received only 0.2% (165) of all grants. Almost three-quarters of all grants were for non-communicable diseases (72%; 40,035), followed by communicable, maternal, perinatal and nutritional conditions (20%; 11,123), and injuries (6%; 3056). Only 1.1% of grants were for neglected tropical diseases and 0.4% for priority diseases on the WHO list of highly infectious (R&D blueprint) pathogens.

**Conclusions:**

The findings highlight the importance of considering funding decisions by other actors in future health research and capacity-strengthening decisions. This will not only improve efficiency and equity in allocating scarce resources but will also allow informed investment decisions that aim to support research on public health needs and neglected areas.

## Background

For the first time, data from major funders of biomedical research are collated in a harmonised and standardised way through the World RePORT platform, allowing for instrumental information on what is being funded, by whom and where, to be analysed and shared on a yearly basis and on a global level. The availability of this information fills an important knowledge gap where this type of information was only available for some diseases or countries [1–6].

The World RePORT platform is hosted by the United States of America’s National Institutes of Health (NIH) and represents a coordinated and collaborative data-sharing effort among 10 major funders of health research that are members of the Heads of International Research Organizations group [7]. Collectively, 8 of the 10 funders that have reported since 2012 account for approximately 76% of the annual health research expenditure of 41 major public and philanthropic funders of health research, as reported by Viergever and Hendriks in 2015 [8].

The specific objectives of this study are to explore how investment decisions on biomedical research by the 10 funders who reported data in 2016 have been allocated among recipient countries and organisations and to develop a method using text data-mining techniques to classify these grants into health categories. This analysis allows the assessment of what is being funded more broadly and for particular health areas of global importance such as research grants for neglected diseases and for pathogens on the research and development (R&D) blueprint list, which have been identified by WHO as a priority list of pathogens due to their expected highly infectious nature [9, 10].

This analysis is part of the World Health Organization Global Observatory on Health Research and Development work’s with the overall goal of enabling evidence-informed deliberations and decisions on priorities for new investments in health R&D [11].

## Methods

### Data source

Grants data for 2016 were collected using the export function of the World RePORT online platform, complemented, where available, with grant abstracts collected directly from each funder’s website and mapped to the exported World RePORT database using the unique grant identifier number.

The World RePORT data include information on direct (primary) grants provided to recipient institutions as well as collaborations with other institutions resulting from these grants (indirect grants administered by recipient institutions).

### Data analysis

The analysis first explored the distribution of direct grants according to the parameters below and then explored the nature of collaborations between institutions that resulted from those direct grants. The following questions were explored (the analysis is also available in interactive data visualisations from the WHO Global Observatory for health R&D, which enables exploration of several of these parameters in relation to each other [12, 13]):

1. Distribution of grants by:
  - funder
  - grant recipients’ region, income group, country and institutions
  - type of grant (e.g. research, training)
  - health category: disease or condition
2. Average grant duration
3. Nature of collaborations between recipients of direct grants and institutions they collaborated with

The data on funding amounts for 2016 was also explored but, since they have not been complete or harmonised yet for 2016, they were not considered for this analysis.

Data checks for consistency and internal validity were performed using Microsoft Excel software. These included internal validity such as valid range of years or uniform country names.

### Classification of grants by region and income group

Regional classification follows the WHO regional groupings [14]. Country income group classification is based on the world development indicators of the World Bank [15]. When the country or area was not included in the World Bank income classification list (2% of the data), we performed an online search of the most recent and reliable data on gross domestic product per capita for these areas and applied the cut-off point for income groupings proposed by the World Bank to classify them into one of the four income groups [16].

### Classification of grants by type

To determine the type of grant, we searched for existing taxonomies, glossaries or categories of the type of grants from the websites of major health research funders (such as National Science Foundation’s glossary and NIH’s glossary and acronym list) and contacted the focal points of each the World RePORT platform funders for any unpublished sources. The lists we retrieved generally included long lists of keywords not appearing to belong to an intentional classification of projects by type (e.g. outcomes, software, database, evaluation, anthropology). We therefore developed our own synonyms list to capture the various terms used to refer to the following categories that emerged from the data: core institutional funding, training (e.g. postgraduate degrees), capacity strengthening (e.g. fellowship, prize), meetings and networking. All other grants falling outside of these categories were classified as research. The categories and list of synonyms for each category were refined and expanded in various iterations during data cleaning and analysis. This was done by reviewing the grant titles and searching for various ways of expressing the category in a snowball manner, including language variations. The search continued until no further synonyms were found.

### Classification of grants by health category

Automated data-mining techniques were used to classify grants by health category. JavaScript and Microsoft Excel were used for this analysis.

First, a comprehensive list of disease synonyms was compiled using the following sources: the Unified Medical Language System, the 10th version of the International Classification of Diseases (ICD-10) and the WHO Global Health Estimates disease list. The list was then complemented by synonyms found in the text fields (titles, keywords, abstracts) of the various databases used by the WHO Global Observatory on Health R&D such as the WHO International clinical trials registry platform, the World RePORT and the AdisInsight database for product pipeline analysis [17–19]. The list also includes abbreviations or language variations as well as misspellings.

Next, a code for an automated algorithm to classify the grants into health categories was written in Java to screen two textual data fields, the grant’s title and the abstract for a match with the synonyms list. The algorithm was constructed to screen the title first; if a match was found, the algorithm stopped, if not, the abstract field was searched next. The algorithm stopped when the first match closer to the beginning of the text field was found. This avoids the assignment of more than one disease. This method was developed and verified using at least five random samples of 100 records from the data to test and refine the comprehensiveness of the synonyms list, which confirmed that the primary disease focus of the grant was almost always the one first mentioned in the text-based field. This was particularly consistent in the title field. It is possible that a grant has more than one disease focus; this is not captured by this algorithm, but its significance (frequency of occurrence) was tested in the sensitivity analysis.

#### Sensitivity analysis for the health category classification approach

To assess the accuracy of the disease categorisation algorithm, we first stratified the data by funder and calculated the percentage of each funder’s contribution to the total number of direct grants in 2016. We then drew a random sample aiming for 100 records, representing a confidence level of 95%. The sample was weighted by funders contribution, which after rounding up, resulted in 107 records. Indirect grants (resulting from collaborations with primary grant recipients) were excluded from this analysis as they had the same title and abstract as direct grants. The sample was drawn from the whole data, whether ultimately classified or not.

Two authors independently reviewed the sample (AHR and TA). At the end of the process, the coding by reviewers was compared, and any discrepancy was resolved by consensus. The following process was used:

- If a classification was available, record (yes or no) whether the disease categorisation is accurate
- For inaccurate or no classification, classify the reasons into the following categories:
  - Use of unspecific or highly technical language without reference to a disease (e.g. molecular biology, cell biology, biochemistry, basic sciences)
  - General topics with no disease focus, including non-research types of grants such as training or core funding
  - New synonyms discovered
  - The disease was not the first mentioned close to the beginning of the text field
  - The topic of the grant was on more than one disease

## Results

### Distribution of grants by funder, type of grant and average grant duration

**Fig. 1:**
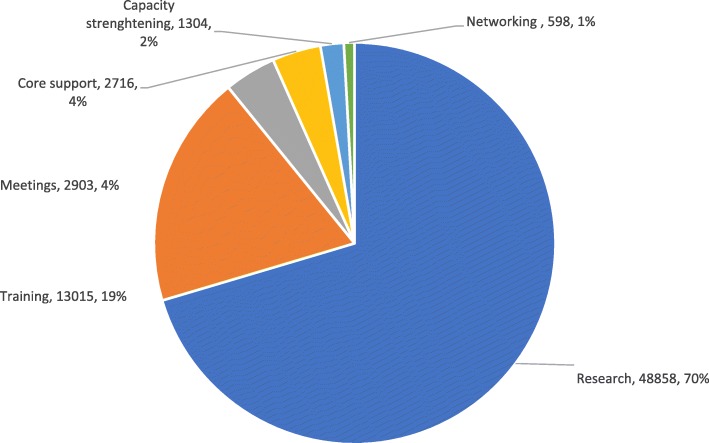
Distribution of biomedical grants in 2016 by type

As shown in Table 1, a total of 69,420 grants were provided by the 10 funding organisations in 2016. The United States of America’s NIH funded the greatest number of grants (52,928; 76%) and had the longest average grant duration (6 years and 10 months). Out of the total number of grants, 70.4% were for research (48,879), followed by training (13,008; 18.7%) and meetings (2907; 4.2%) (Fig. 1).

**Table 1:** Distribution of grants for biomedical research and average grant duration by funder in 2016

Funding organisation	Number of grants in 2016	Average grant duration
National Institutes of Health (NIH)	52,928	6 years, 10 months
Canadian Institutes of Health Research (CIHR)	5567	4 years, 4 months
Wellcome Trust	5273	3 years, 8 months
Medical Research Council (MRC)	2649	4 years, 7 months
European Commission (EC)	1076	2 years, 11 months
Swedish Research Council (SRC)	999	3 years, 7 months
Bill & Melinda Gates Foundation (BMGF)	783	3 years, 7 months
Institut Pasteur	99	1 years, 6 months
Swedish International Development Cooperation Agency (Sida)	25	2 years, 11 months
European & Developing Countries Clinical Trials Partnership (EDCTP)	21	2 years, 9 months
Total	69,420

### Distribution of grants by recipients’ region, income group, country and institution

Of grant recipients by income group, high-income countries received 98.9% of all grants, whereas low-income countries received only 0.2% (165) (Table 2). Among the 450 grants received by African countries (Table 3), South Africa (upper–middle-income country) received the highest number of grants (156; 34.7%) and was the fifth on the list of top 10 countries that received the highest number of grants. The remaining 9 countries were in the European (7) and the Americas regions (2) (Table 3).

**Table 2:** Distribution of biomedical grants in 2016 by WHO region and income group

WHO region	High income	Upper–middle income	Lower–middle income	Low income	Unspecified	Grand total
Africa		165	124	161		450
Americas	58,720	75	15	1		58,811
Eastern Mediterranean		7	12	1		20
Europe	9854	11	3			9868
South-East Asia		13	107	2		122
Western Pacific	86	38	24			148
Multiple regions					1	1
Grand total	68,660	309	285	165	1	69,420

**Table 3:** Top 10 recipient countries and top recipient institution within each country (2016)

No.	Country name	Number of grants	WHO Region	Income group	Top recipient institution	Number of grants
1	United States of America	53,114	Americas	High	Johns Hopkins University	1314
2	United Kingdom	7642	Europe	High	University of Oxford	978
3	Canada	5576	Americas	High	University of British Columbia	677
4	Sweden	1055	Europe	High	Karolinska Institutet	435
5	South Africa	156	Africa	Upper–middle	University of Cape Town	62
6	Germany	143	Europe	High	Max Planck Society for the Advancement of Science	13
7	Ireland	137	Europe	High	University College Dublin	48
8	France	134	Europe	High	Institut national de la santé et de la recherche médicale (Inserm), Paris	21
9	Netherlands	122	Europe	High	Stichting katholieke univeriteit (catholic university foundation)	11
10	Switzerland	111	Europe	High	World Health Organization	19

### Distribution of grants by health category

**Fig. 2:**
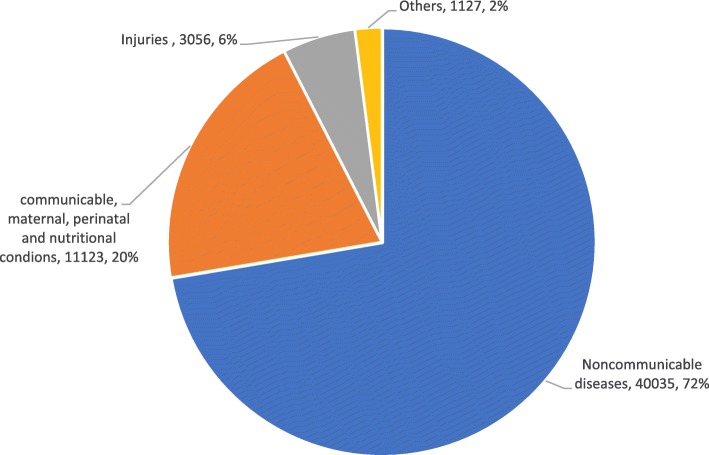
Distribution of biomedical grants in 2016 by health category

Almost three-quarters of all grants were for non-communicable diseases (72%; 40,035), followed by communicable, maternal, perinatal and nutritional conditions (20%; 11,123) and injuries (6%; 3056) (Table 4, Fig. 2).

**Table 4:** Top health categories, subcategories and diseases/conditions funded in 2016

Health category	Number (%)	Top four health subcategories	Number (%)	Top diseases/conditions within each subcategory	Number (%)
Non-communicable	40,035 (72%)	Malignant neoplasms	9483 (24%)	Breast cancer	803 (8%)
		Mental and substance use disorders	5945 (15%)	Alcohol use disorders	574 (10%)
		Neurological conditions	4981 (12%)	Alzheimer disease and other dementia	1792 (36%)
		Cardiovascular diseases	4473 (11%)	Stroke	632 (14%)
Communicable, maternal, perinatal and nutritional conditions	11,123 (20%)	Infectious and parasitic diseases	8826 (79%)	HIV/AIDS	3039 (34%)
		Respiratory infections	738 (7%)	Lower respiratory infections	616 (83%)
		Nutritional deficiencies	651 (6%)	Protein/energy malnutrition	488 (75%)
		Neonatal conditions and maternal conditions	496 (4%)	Birth asphyxia and birth trauma	200 (40%)
Injuries	3056 (6%)	Injury, poisoning and certain other consequences of external causes	2776 (91%)	Injuries to unspecified part of trunk, limb or body region	1242 (45%)
		External causes of injuries	280 (9%)	Self-harm	112 (40%)
Others	1127 (2%)

Among non-communicable diseases, 24% (9483 grants) were for malignant neoplasms, followed by mental and substance use disorders (15%; 5945), neurological conditions (12%; 4981), and cardiovascular diseases (11%; 4473). Among communicable, maternal, perinatal and nutritional conditions, nearly 80% of grants (8826) were for infectious and parasitic diseases, followed by respiratory infections (7%; 738), nutritional deficiencies (6%; 651) and neonatal conditions and maternal conditions (both at 4%; 496 and 412, respectively) (Table 4).

Looking at select health areas of global importance, analysis of grants for neglected tropical diseases show that they represented 1.1% (792) of all grants, of which dengue (16%; 125 grants) and leishmaniasis (13%,102 grants) were the two individual diseases that received the highest number of grants. Similarly, 0.4% (274) of all grants were for one of the priority diseases on the WHO list of highly infectious pathogens (R&D blueprint pathogens); 83% of these were for Ebola virus disease (43%; 117), Zika virus disease (32%; 89) and severe acute respiratory syndrome (8%; 21).

### Nature of collaborations resulting from direct grants

Around 10% (6918) of direct grants resulted in collaborations with other institutions, which did not always translate into a transfer of funds from the primary recipient to the collaborating institutions; 96.4% (6669) of these direct grants had been awarded to recipients in high-income countries (Table 5) and 75.8% (14,619) of the collaborations resulting from these grants were with others in high-income countries. In fact, for each income group, collaborations were most likely to be with others in the same income group, followed by institutions in high-income countries. For example, grant recipients in low-income countries (66) collaborated most with institutions in low-income countries (88), followed by institutions in high-income (78), lower–middle-income (11) and upper–middle-income (8) countries (Table 5).

**Table 5:** Distribution of collaborations between direct grant recipients and collaborating institutions by income group in 2016

Direct grant recipient country’s income group	Number of direct grants	Collaborating institution country’s income group	Number of collaborations
High	6669	High	14,364
		Upper–middle	2041
		Lower–middle	1338
		Low	956
Upper-middle	105	Upper–middle	232
		High	103
		Lower–middle	34
		Low	16
Lower-middle	78	Lower–middle	101
		High	84
		Upper–middle	12
		Low	15
Low	66	Low	88
		High	78
		Lower–middle	11
		Upper–middle	8
Grand total	6918		19,283

Note: One direct grant can result in multiple collaborations. Various combinations and details (countries, institutions and diseases involved) of this analysis can be explored interactively on the Observatory’s website [12]

### Sensitivity analysis

Table 6 describes the sample size for the sensitivity analysis and the percentage of each funder’s contribution to the total number of direct grants (69,420) in 2016. The sample consisted of 107 records, after rounding up of percentage figures.

**Table 6:** Sample size for the sensitivity analysis of the disease classification method

Funding organisation	Total number of grants	Contribution to sample	Sample size
National Institutes of Health (NIH)	52,928	76.24%	77
Canadian Institutes of Health Research (CIHR)	5567	8.02%	9
Wellcome Trust	5273	7.60%	8
Medical Research Council (MRC)	2649	3.82%	4
European Commission (EC)	1076	1.55%	2
Swedish Research Council (SRC)	999	1.44%	2
Bill & Melinda Gates Foundation (BMGF)	783	1.13%	2
Institut Pasteur	99	0.14%	1
Swedish International Development Cooperation Agency (Sida)	25	0.04%	1
European & Developing Countries Clinical Trials Partnership (EDCTP)	21	0.03%	1
Total	69,420	100.00%	107

Table 7 shows that, out of a random sample of 107 grants, 81% were assigned to a health category and, in 91% of the cases, the classification was accurate. Classification accuracy was 98% when the title was used compared to 84% when the abstract was used. However, classification based on abstract contributed around 50% of classified grants, hence its usefulness. In 40% of the cases when a grant was not classified, no abstract was available. In the 28 cases where grants were misclassified, the main reasons were unspecific or very technical language used with no disease mentioned (11; 39%), general topic not linked to a specific disease focus (7; 25%), or new synonyms were discovered that could have allowed a classification to be made (9; 32%).

**Table 7:** Results of the sensitivity analysis for the disease classification method (sample = 107)

Element of the analysis	Number	Percentage
A disease classification was attributed	87/107	81%
Accuracy of the results		
General	79/87	91%
Based on the grant’s title	42/43	98%
Based on the grant’s abstract	37/44	84%
Among misclassified or unclassified grants		
Primary disease was not mentioned first	1/28	4%
Unspecific or highly technical language used with no disease mentioned	11/28	39%
General topics with no specific disease focus	7/28	25%
New synonyms discovered that were not included	9/28	32%

Overall, applying a data-mining algorithm that selects the first mention of a disease in the title or, failing this, the abstract, appears to yield reliable results; only in 1% of all classified grants (1/87) was the primary disease not the first mentioned in the title or abstract. In this case, the attributed disease was associated with the primary disease topic of the research.

## Discussion

The analysis presented in this paper provides, for the first time, an overall overview of what is being funded, by whom and where, among major international funders of biomedical research globally and for all disease areas.

The analysis highlights important findings on current resource allocation decisions and the nature and reach of research collaborations across regions. These include the large share (72%) of non-communicable diseases among all grants, the very small proportion of direct funding reaching low-income countries (0.2%), and the fact that neglected diseases such as those on the WHO list of neglected tropical diseases remain very neglected in terms of R&D investments (only 1.1% of all grants provided to this area) [10].

These findings are consistent with a recent analysis of health products in the pipeline from discovery to market launch for all diseases globally, which showed that 87% of products are for non-communicable diseases and less than 0.5% where for one of the diseases on the WHO list of neglected tropical diseases [20].

Additional details and a multitude of iterations and combinations of the analysis presented in this paper can be explored on the WHO Global Observatory on Health R&D website, allowing for various combinations of questions to be examined together (by funder, disease, institution, etc.) [12, 13].

This information will help funders of health research explore how best to increase efficiency, coordinate investments, contribute to capacity for health research and focus on areas where there are needs and gaps. It is also of interest to researchers to explore areas where research gaps or abundancies exist among these funders, topic areas of interest and expertise among research institutions for possible future collaborations as well as main areas of interest for these funders.

The Observatory will continue to update this analysis with new data, which will allow, over time, an analysis of trends in research allocation and collaborations to be explored, including the extent to which research funding for areas where public health needs of low- and middle-income countries are greatest are covered and the extent to which research institutions in these countries are benefiting from these grants.

This paper also made an important contribution to automated data-mining methodologies applied to health data by developing and testing the hypothesis that the primary disease focus of a submission is most likely be the first-mentioned closest to the beginning of the text field. The fact that this was also applicable to the abstract is very encouraging, as almost 50% of the grants were classified using the abstract field, allowing a higher proportion of the grants to be classified. That said, the title was the most accurate field for textual data mining when it was comprehensively written.

Overall, and considering the results of the sensitivity analysis, this method provides a reasonable solution to categorise and analyse a multitude of databases by health category – this is important information for monitoring and setting priorities for new investments in health research and development. The health category and synonyms list are available on the Observatory website and will be periodically updated with new synonyms to encourage further data analysis and knowledge-sharing in this field [21].

As with any analysis of this type, various limitations are involved, including the small number number of funders included, the likelihood that the classification of grants by category and type did not accurately classify grants, and the fact that some funders were not able to account for all the collaborations resulting from their primary grants due to lack of information on these.

That said, the funders included in this analysis are estimated to contribute a high proportion of annual investments in health research globally [8], and the results of the sensitivity analysis of the data-mining method yielded very encouraging results. Therefore, these findings can be considered a reasonable indication of what is being funded by these funders and can serve as a basis for the expansion of this analysis and further improvement in funder and research grant databases. Most importantly, the findings presented here provide various insights on important resource allocation questions that we hope will assist in informing future investment decisions.

Areas for improvement in the development and maintenance of research grant databases include making available a health category field, ideally using a drop-down menu to avoid the inhomogeneous entries of text fields, that the applicants can use to categorise their submission as well as a field to categorise the type of grant into the research (with their subcategories) or non-research categories, which would tremendously contribute to the better coordination and monitoring of capacity-strengthening initiatives worldwide.

## Conclusion

The findings presented here provide a cross-sectional view of investment decisions by 10 major international funders of health research, whose value extends beyond the actual information presented here to further stimulating the thinking about key elements, trends and tendencies in global resource allocation for R&D in general. More importantly, it highlights the persistent low investments for important public health areas such as neglected diseases (1.1%) and the very small share of international research funding going to low-income countries (0.2%). The findings, and the various other combinations of questions that can be explored through the Observatory’s data visualisations, provide new knowledge and insights as well as endless possibilities to test different patterns and relationships for all diseases or R&D areas, thus maximising the potential of learning from available data that was previously unexploited.

## Data Availability

Data generated or analysed during this study can be obtained from the World RePORT (https://worldreport.nih.gov/) and are available on the WHO Global Observatory on health R&D (the Observatory), at: http://www.who.int/research-observatory/en/. Future analysis, updates and expansions of the analysis in this study will be available on the WHO Observatory.

## Abbreviations

NIH: National Institutes of Health
R&D: Research and development
